# The exact solutions and approximate analytic solutions of the (2 + 1)-dimensional KP equation based on symmetry method

**DOI:** 10.1186/s40064-016-2908-8

**Published:** 2016-08-05

**Authors:** Litao Gai, Sudao Bilige, Yingmo Jie

**Affiliations:** 1 College of Sciences, Inner Mongolia University of Technology, Hohhot, 010051 China; 2 College of Sciences, Dalian University of Technology, Dalian, 116024 China

**Keywords:** Symmetry, Wu-differential characteristic set algorithm, The extended tanh method, The homotopy perturbation method

## Abstract

In this paper, we successfully obtained the exact solutions and the approximate analytic solutions of the (2 + 1)-dimensional KP equation based on the *Lie* symmetry, the extended tanh method and the homotopy perturbation method. In first part, we obtained the symmetries of the (2 + 1)-dimensional KP equation based on the Wu-differential characteristic set algorithm and reduced it. In the second part, we constructed the abundant exact travelling wave solutions by using the extended tanh method. These solutions are expressed by the hyperbolic functions, the trigonometric functions and the rational functions respectively. It should be noted that when the parameters are taken as special values, some solitary wave solutions are derived from the hyperbolic function solutions. Finally, we apply the homotopy perturbation method to obtain the approximate analytic solutions based on four kinds of initial conditions.

## Background

Recently, the nonlinear phenomenon has been extensively appeared in the fields of mathematical physics and engineering technology. A multitude of research focuses have been changed from linear problems to nonlinear ones. These problems can be ascribed to the research of nonlinear partial differential equations (NLPDE), as the complexity of equation, it becomes hard to get the exact solutions. Hence, the investigation of solving NLPDE has important theoretical and practical significance. In recent decades, a growing number of scholars established effective methods and these methods have obtained comprehensive applications. Such as the symmetry method (Lie 1881), the CK direct method (Clarkson and Kruskal 1989), the homogeneous balance method (Wang and Li 1996), the tanh-function method (Fan 2000; Akbar et al. 2013; Xie et al. 2005), the F-expansion method (Wang and Li 2005), sub-ODE method (Zhang et al. 2006), the simplest equation method (Kudryashov 2005; Sudao and Temuer 2010), the $(G^{\prime }/G)$-expansion method (Wang et al. 2008; Alam and Akbar 2013), the homotopy perturbation method (He 1999; Narayanamoorthy and Sathiyapriya 2016; Filobello-Nino et al. 2016) and so on. However, a unified and systemic method, which can be applied to solve all kinds of equations, is still inexistence, and the above-mentioned methods all have individual range of applications. Therefore, summarizing and concluding, adopting the advantages and abandoning the disadvantages have been regarded as the effective approaches to investigate these existing methods. At the same time, it is worth obtaining more new solutions of NLPDE by using *Lie* symmetry and other methods.

As we all know, the symmetry method is the most universal method, and many traditional methods become its special cases. During the end of the nineteenth century, in order to unify and expand the methods used in solving the ordinary differential equations(ODE), Norwegian mathematician *Sophus Lie* (1842–1899) firstly proposed the symmetry theory of differential equations (Lie 1881). The investigations of the symmetry theory and approach have important theoretical and practical significance in modern mathematics, physics, mechanics and so on, at the same time, many successful applications have emerged in those fields (Bluman and Kumei 1989; Bluman et al. 2009; Noether 1918; Ma 1990; Clarkson and Kruskal 1989; Lou and Tang 2001; Ma and Chen 2009; Ma 2013). At present, using the symmetry method and others, such as the analytic solutions method, the approximate analytic solutions method and the numerical method with the aid of thorough considering mutual complementarity and availability to solve NLPDE are the new research subjects.

The premise of applying the symmetry method is to determine the all kinds of symmetries of the partial differential equations (PDEs). The main approach of determining the symmetries is the infinitesimal transform method which is proposed and constructed by *Lie*, called *Lie*’s algorithm. *Lie*’s algorithm, which is the major method with respect to determining symmetries, transforms the problem of determining symmetries into that of determining corresponding infinitesimal vectors whose infinitesimal functions are found as solutions of some over-determined system of PDEs, called the determining equations (Lie 1881). In determining symmetries, tedious, mechanical computations are involved and the order relation of unknown quantities have not been considered in conventional *Lie*’s algorithm, which result many problems, such as infinite loops on computers, a mass of work and so on. According to the investigations, differential form Wu’s method is one of effective methods to get rid of the defects of *Lie*’s algorithm. Therefore, Wu-differential characteristic set algorithm extended and constructed by Temuer Chaolu can partially solve the above-mentioned problems (Temuer 1999; Temuer and Bai 2010). This algorithm has been successfully applied to classical symmetries, nonclassical symmetries, high-order symmetries, approximate symmetries, potential symmetries, conservation laws and symmetry classification of PDEs, which has promoted the investigations of symmetry theory of PDEs (Bluman and Temuer 2006; Temuer et al. 2007; Temuer and Bai 2009; Temuer and Pang 2010; Sudao et al. 2014). Recently, we investigate the applications of the symmetry method in the boundary value problem of the nonlinear PDEs based on Wu-differential characteristic set algorithm and use the symmetry method and the homotopy analytic method to solve the boundary value problem (Sudao et al. 2014; Sudao 2011). Some other investigators use the symmetry method, the variational iterative method and the homotopy perturbation method to solve the boundary value problem based on Wu-differential characteristic set algorithm (Lu and Temuer 2011a, b; EerDun and Temuer 2012).

In this paper, we will construct the exact solutions and the approximate analytic solutions of the (2 + 1)-dimensional KP equation by using the *Lie* symmetry, the extended tanh method and the homotopy perturbation method. The Wu-differential characteristic set algorithm plays an important role in calculating the symmetries of the (2 + 1)-dimensional KP equation. This investigation will explore a new approaches of *Lie* symmetry in application of NLPDE. In addition, it will also effectively popularize the range of application and advance the efficiency of using method.

## The symmetries and symmetry reduction of the (2 + 1)-dimensional KP equation

We consider the (2 + 1)-dimensional KP equation (Ding and Ji 2008) as follow:

1 $$ { u_{xt}-6u_{x}^{2}-6uu_{xx}+u_{xxxx}+3u_{yy}=0} $$

it is applied to describe the law of motion of water waves in (2 + 1)-dimensional spaces as well as plasmas in magnetic fields. Next, we will give the process of calculating the symmetry and reduction of Eq. (1).

### The symmetries of the (2 + 1)-dimensional KP equation

The symmetry group of Eq. (1) will be generated by the vector field of the form

2 $$ X=\xi (x,t,y,u)\frac{\partial }{\partial x}+\mu (x,t,y,u)\frac{\partial }{\partial t} +\tau (x,t,y,u)\frac{\partial }{\partial y}+\eta (x,t,y,u)\frac{\partial }{\partial u} $$

where $\xi$, $\mu$, $\tau$, $\eta$ are the infinitesimal generated functions of the symmetry. According to the *Lie* algorithm, we obtain the determining equations of symmetry (2), but it is too difficult to get its solutions. However, we can obtain the followig system of equations corresponding to the characteristic set which is equivalent to the determining equations by using Wu-differential characteristic set algorithm (Temuer 1999).

$$\begin{aligned}&\xi _{xx}=\xi _{xy}=\xi _u=0,\quad \mu _x=\mu _{y}=\mu _u=0,\quad \tau _x=\tau _u=0,\\& \quad \eta _{xx}=\eta _{xy}=0,\quad 3\eta _{yy}+\eta _{xt}=0,\quad 2\eta _x-\xi _{yy}=0,\quad \mu _t-3\xi _x=0,\\&\quad \eta _u+2\xi _x=0,\quad 6\eta +\xi _t+12u\xi _x=0,\quad 2\xi _x-\tau _y=0,\quad 6\xi _y+\tau _t=0 \end{aligned}$$

By solving the above PDEs, we get the infinitesimal functions

3 $$\xi =c_1x-3c_4t^2+c_5,\quad \mu =3c_1t+c_2, \quad \tau =2c_1y+c_3,\quad \eta =c_4t-2c_1u $$

where $c_1$, $c_2$, $c_3$, $c_4, c_5$ are arbitrary constants, then the corresponding infinitesimal vector is the following form

4 $$ X=(c_1x-3c_4t^2+c_5)\frac{\partial }{\partial x}+(3c_1t+c_2)\frac{\partial }{\partial t} +(2c_1y+c_3)\frac{\partial }{\partial y}+(c_4t-2c_1u)\frac{\partial }{\partial u}$$

Obviously, *X* has five one-parameter point symmetries, then the corresponding infinitesimal vectors are as follow:

5 $$\begin{aligned}&X_1=x\frac{\partial }{\partial x}+3t\frac{\partial }{\partial t}+2y\frac{\partial }{\partial y}-2u\frac{\partial }{\partial u}, \quad X_2=\frac{\partial }{\partial t}, \quad X_3=\frac{\partial }{\partial y},\nonumber \\&X_4=-3t^2\frac{\partial }{\partial x}+t\frac{\partial }{\partial u},\quad X_5=\frac{\partial }{\partial x} \end{aligned}$$

### The reduction of Eq. (1)

To facilitate solve the Eq. (1), we will reduce it by using the invariant form method. The resulting reduced PDE is fewer independent variable than Eq. (1).

#### *Case 1*

When $\chi _1$ = $X_1$, we obtain $u(x,t,y)=U[\xi _1,\xi _2]/x^2$ by solving the characteristic equation ${\frac{dx}{x}}={ \frac{dt}{3t}}={\frac{dy}{2y}}={ \frac{du}{-2u}}$, where $\xi _1=x^3/t$, $\xi _2=x^2/y$ are the invariants, then the reduction of Eq. (1) is

6 $$\begin{aligned} &40U-20U^2-40\xi _2U_2+2\xi _2^3U_2+28\xi _2UU_2-8\xi _2^2U_2^2+20\xi _2^2U_{22}+\xi _2^4U_{22}-8\xi _2^2UU_{22}\\&\quad -\frac{16}{3}\xi _2^3U_{222}+\frac{16}{3}\xi _2^4U_{2222}-40\xi _1U_1-\frac{1}{3}\xi _1^2U_1+36\xi _1UU_1-24\xi _1\xi _2U_1U_2-18\xi _1^2U_1^2\\&\quad +40\xi _1\xi _2U_{12}-24\xi _1\xi _2UU_{12}+32\xi _1\xi _2^3U_{1222}+24\xi _1^2U_{11}-\xi _1^3U_{11}-18\xi _1^2UU_{11}\\&\quad -\frac{2}{3}\xi _1^2\xi _2U_{12}+36\xi _1^2\xi _2U_{112}+72\xi _1^2\xi _2^2U_{1122}+36\xi _1^3U_{111}+72\xi _1^3\xi _2U_{1112}+27\xi _1^4U_{1111}=0 \end{aligned} $$

By the same token, we will get the following reductions.

#### *Case 2*

When $\chi _2$ = $X_2+X_3$, we reduce to Eq. (1) by using the invariant form method as follow:

7 $$ U_{12}-3U_{22}+6U_1^2+6UU_{11}-U_{1111}=0 $$

where $u(x,t,y)=U[\xi _1,\xi _2]$, and $\xi _1=x$, $\xi _2=y-t$ are the invariants.

#### *Case 3*

When $\chi _3$ = $X_2+X_5$, we reduce to Eq. (1) as follow:

8 $$ U_{11}-3U_{22}+6U_1^2+6UU_{11}-U_{1111}=0 $$

where $u(x,t,y)=U[\xi _1,\xi _2],$ and $\xi _1=x-t$, $\xi _2=y$ are the invariants.

#### *Case 4*

When $\chi _4$ = $X_2+X_4$, we reduce to Eq. (1) as follow:

9 $$ 3U_{22}-6U_1^2-6UU_{11}+U_{1111}=0 $$

where $u(x,t,y)=U[\xi _1,\xi _2]+t^2/2$, and $\xi _1=x+t^3$, $\xi _2=y$ are the invariants.

#### *Case 5*

When $\chi _5$ = $X_3+X_5$, we reduce to Eq. (1) as follow:

10 $$ U_{12}+3U_{22}-6U_2^2-6UU_{22}+U_{2222}=0 $$

where $u(x,t,y)=U[\xi _1,\xi _2]$, and $\xi _1=t$, $\xi _2=x-y$ are the invariants.

#### *Case 6*

When $\chi _6$ = $X_3+X_4+X_5$, we reduce to Eq. (1) as follow:

11 $$U_{11}+U_{12}-3U_{22}+6U_1^2 +6UU_{11}-U_{1111}=0 $$

where $u(x,t,y)=U[\xi _1,\xi _2]$, and $\xi _1=x-t$, $\xi _2=y-t$ are the invariants.

In all above, $$U_i=\frac{\partial U}{\partial \xi _i}$$, $$U_{ij}=\frac{\partial U^2}{\partial \xi _i\partial \xi _j}$$, $$(i, j=1, 2)$$, such as $$U_{11}=\frac{\partial U^2}{\partial \xi _1\partial \xi _1}$$, $$U_{12}=\frac{\partial U^2}{\partial \xi _1\partial \xi _2}$$, $$U_{112}=\frac{\partial U^3}{\partial \xi _1\partial \xi _1\partial \xi _2}$$$$\ldots $$ and so on. From the above Eqs. (6) to (11), it is not difficult to find that Eq. (1) is reduced into the variable coefficient equations by using the symmetry $$\chi _1$$ and the constant coefficient equations by using the symmetries $$\chi _2$$–$$\chi _6,$$ respectively.

## The exact travelling wave solutions of (8) based on the extended tanh method

Recently, as an effective approach, the extended tanh method is introduced to seek the exact solutions of the nonlinear evolution equations by Xie et al. (2005). This method is further improved by the generalized Riccati equation and introducing its twenty seven new solutions, these solutions are expressed by the hyperbolic functions, the trigonometric functions and the rational functions, respectively. When the parameters are taken as special values, some solitary wave solutions are derived from the hyperbolic function solutions.

Taking Eq. (8) for example from the symmetry reduction equations, we will get its exact travelling wave solutions by the extended tanh method and the process is composed of the following four steps.

### *Step 1*

 Doing the travelling wave transformations. In order to look for the travelling wave solutions of Eq. (8), we introduce the travelling wave transformation as follows:

12 $$ U(\xi _1,\xi _2)=U(\xi ),\quad \xi =k\xi _1-c\xi _2 $$

where *k*, *c* are constants and $\xi _1=x-t, \xi _2=y$. Then we reduce Eq. (8) into ODE for $U(\xi )$, namely

13 $$\left( 3c^2-k^2\right) U^{\prime \prime }-6k^2\left( U^{\prime }\right) ^2-6k^2UU^{\prime \prime }+k^4U^{(4)}=0 $$

### *Step 2*

 Choosing the expression of solution. By considering the homogeneous balance between the highest order derivatives $U^{(4)}$ and nonlinear terms $UU^{\prime \prime }$ appearing in Eq. (13), we choose the following expression of solution:

14 $$ U=\alpha _0+\alpha _1\phi +\alpha _2\phi ^2 $$

where $\alpha _0$, $\alpha _1$, $\alpha _2$ are undetermined coefficients. The function $\phi =\phi (\xi )$ satisfies the second-order linear ODE

15 $$ \phi ^{\prime }=\lambda +\delta \phi +\nu \phi ^2 $$

where $\lambda$, $\delta$, $\nu$ are constants. The ODE (15) has four cases of solutions as follows.

### *Case 1*

When $\delta ^2-4\lambda \nu >0$ and $\delta \nu \ne 0$ (or $\nu \lambda \ne 0$),

$$\begin{aligned}&\phi _{1}=-\frac{1}{2\nu }\left( \delta +\sqrt{\theta }\tanh \left[ \frac{\sqrt{\theta }}{2}\xi \right] \right) ,\quad \phi _{2}=-\frac{1}{2\nu }\left( \delta +\sqrt{\theta }\coth \left[ \frac{\sqrt{\theta }}{2}\xi \right] \right) \\&\phi _{3}=-\frac{1}{2\nu }\left( \delta +\sqrt{\theta }\left( \tanh \left[ \sqrt{\theta }\xi \right] \pm i\mathrm{sech}\left[ \sqrt{\theta }\xi \right] \right) \right) ,\\&\phi _{4}=-\frac{1}{2\nu }\left( \delta +\sqrt{\theta }\left( \coth \left[ \sqrt{\theta }\xi \right] \pm i\mathrm{csch}\left[ \sqrt{\theta }\xi \right] \right) \right) \\&\phi _{5}=-\frac{1}{4\nu }\left( 2\delta +\sqrt{\theta }\left( \tanh \left[ \frac{\sqrt{\theta }}{4}\xi \right] +\coth \left[ \frac{\sqrt{\theta }}{4}\xi \right] \right) \right) \\&\phi _{6}=\frac{1}{2\nu }\left( -\delta +\frac{\sqrt{\left( A^2+B^2\right) \theta }-A\sqrt{\theta }\cosh \left[ \sqrt{\theta }\xi \right] }{A\sinh \left[ \sqrt{\theta }\xi \right] +B} \right) \\&\phi _{7}=\frac{1}{2\nu }\left( -\delta -\frac{\sqrt{\left( B^2-A^2\right) \theta }+A\sqrt{\theta }\sinh \left[ \sqrt{\theta }\xi \right] }{A\cosh \left[ \sqrt{\theta }\xi \right] +B} \right) \end{aligned}$$

where *A* and *B* are two nonzero constants and satisfies $B^2-A^2>0,$

$$\begin{aligned}&\phi _{8}=\frac{2\lambda \cosh \left[ \sqrt{\theta }\xi /2\right] }{\sqrt{\theta }\sinh \left[ \sqrt{\theta }\xi /2\right] -\delta \cosh \left[ \sqrt{\theta }\xi /2\right] }\\&\phi _{9}=\frac{-2\lambda \sinh \left[ \sqrt{\theta }\xi /2\right] }{\delta \sinh \left[ \sqrt{\theta }\xi /2\right] -\sqrt{\theta }\cosh \left[ \sqrt{\theta }\xi /2\right] }\\&\phi _{10}=\frac{2\lambda \cosh \left[ \sqrt{\theta }\xi \right] }{\sqrt{\theta }\sinh \left[ \sqrt{\theta }\xi \right] -\delta \cosh \left[ \sqrt{\theta }\xi \right] \pm i\sqrt{\theta }}\\&\phi _{11}=\frac{2\lambda \sinh \left[ \sqrt{\theta }\xi \right] }{-\delta \sinh \left[ \sqrt{\theta }\xi \right] +\sqrt{\theta }\cosh \left[ \sqrt{\theta }\xi \right] \pm \sqrt{\theta }}\\&\phi _{12}=\frac{4\lambda \sinh \left[ \sqrt{\theta }\xi /4\right] \cosh \left[ \sqrt{\theta }\xi /4\right] }{-2\delta \sinh \left[ \sqrt{\theta }\xi /4\right] \cosh \left[ \sqrt{\theta }\xi /4\right] + 2\sqrt{\theta }\cosh ^2\left[ \sqrt{\theta }\xi /4\right] - \sqrt{\theta }} \end{aligned} $$

### *Case 2*

When $\delta ^2-4\lambda \nu <0$ and $\delta \nu \ne 0$ (or $\nu \lambda \ne 0$),

$$\begin{aligned} &\phi _{13}=\frac{1}{2\nu }\left( -\delta +\sqrt{-\theta }\tan \left[ \frac{\sqrt{-\theta }}{2}\xi \right] \right) ,\quad \phi _{14}=-\frac{1}{2\nu }\left( \delta +\sqrt{-\theta }\cot \left[ \frac{\sqrt{-\theta }}{2}\xi \right] \right) \\&\phi _{15}=\frac{1}{2\nu }\left( -\delta +\sqrt{-\theta }\left( \tan \left[ \sqrt{-\theta }\xi \right] \pm \sec \left[ \sqrt{-\theta }\xi \right] \right) \right) \\&\phi _{16}=-\frac{1}{2\nu }\left( \delta +\sqrt{-\theta }\left( \cot \left[ \sqrt{-\theta }\xi \right] \pm \csc \left[ \sqrt{-\theta }\xi \right] \right) \right) \\&\phi _{17}=\frac{1}{4\nu }\left( -2\delta +\sqrt{-\theta }\left( \tan \left[ \frac{\sqrt{-\theta }}{4}\xi \right] - \cot \left[ \frac{\sqrt{-\theta }}{4}\xi \right] \right) \right) \\&\phi _{18}=\frac{1}{2\nu }\left( -\delta +\frac{\pm \sqrt{\left( A^2-B^2\right) (-\theta )}-A\sqrt{-\theta }\cos \left[ \sqrt{-\theta }\xi \right] }{A\sin \left[ \sqrt{-\theta }\xi \right] +B} \right) \\&\phi _{19}=\frac{1}{2\nu }\left( -\delta +\frac{\pm \sqrt{\left( A^2-B^2\right) (-\theta )}+A\sqrt{\theta }\sin \left[ \sqrt{\theta }\xi \right] }{A\cos \left[ \sqrt{\theta }\xi \right] +B} \right) \end{aligned} $$

where *A* and *B* are two nonzero constants and satisfies $A^2-B^2>0,$

$$\begin{aligned} &\phi _{20}=\frac{2\lambda \cos \left[ \sqrt{-\theta }\xi /2\right] }{\sqrt{-\theta }\sin \left[ \sqrt{-\theta }\xi /2\right] +\delta \cos \left[ \sqrt{-\theta }\xi /2\right] }\\&\phi _{21}=\frac{-2\lambda \sin \left[ \sqrt{-\theta }\xi /2\right] }{-\delta \sin \left[ \sqrt{-\theta }\xi /2\right] +\sqrt{-\theta }\cos \left[ \sqrt{-\theta }\xi /2\right] }\\&\phi _{22}=\frac{2\lambda \cos \left[ \sqrt{-\theta }\xi \right] }{\sqrt{-\theta }\sin \left[ \sqrt{-\theta }\xi \right] +\delta \cos \left[ \sqrt{-\theta }\xi \right] \pm \sqrt{-\theta }}\\&\phi _{23}=\frac{2\lambda \sin \left[ \sqrt{-\theta }\xi \right] }{-\delta \sin \left[ \sqrt{-\theta }\xi \right] +\sqrt{-\theta }\cos \left[ \sqrt{-\theta }\xi \right] \pm \sqrt{-\theta }}\\&\phi _{24}=\frac{4\lambda \sin \left[ \sqrt{-\theta }\xi /4\right] \cos \left[ \sqrt{-\theta }\xi /4\right] }{-2\delta \sin \left[ \sqrt{-\theta }\xi /4\right] \cos \left[ \sqrt{-\theta }\xi /4\right] +2\sqrt{-\theta }\cos ^2\left[ \sqrt{-\theta }\xi /4\right] -\sqrt{-\theta }} \end{aligned}$$

for the above $\phi _{1}$–$\phi _{24}$, setting $\theta =\delta ^{2}-4\lambda \nu$.

### *Case 3*

 When $$\lambda =0$$ and $$\delta \nu \ne 0,$$

$$\phi _{25}=\frac{-\delta \omega }{\nu \left( \omega +\cosh [\delta \xi ]-\sinh [\delta \xi ]\right) },\quad \phi _{26}=-\frac{\delta \left( \cosh [\delta \xi ]+\sinh [\delta \xi ]\right) }{\nu \left( \omega +\cosh [\delta \xi ]+\sinh [\delta \xi ]\right) } $$

where, $\omega$ is an arbitrary constant.

### *Case 4*

 When $$\nu \ne 0$$ and $$\lambda =\delta =0,$$

$$\phi _{27}=\frac{1}{\nu \xi +c} $$

where *c* is an arbitrary constant, and for the above $\phi _1$–$\phi _{27}$, setting $\xi =k\xi _1-c\xi _2.$

### *Step 3*

Determining the coefficients. By substituting (14) into Eq. (13) and using ODE (15), collecting all terms with the same order of $\phi ^i$ together, the left-hand side of Eq. (13) is converted into another polynomial in $\phi ^i$. Equating each coefficient of this different power terms to zero yields a set of nonlinear algebraic equations for $\alpha _i (i=0,1,2), k, c, \lambda , \delta$ and $\nu$. With the aid of mathematica, we get the solutions as follows:

16 $$\alpha _0=\frac{3c^2-k^2+k^4\delta ^2+8k^4\lambda \nu }{6k^2},\quad \ \alpha _1=2k^2\delta \nu ,\quad \alpha _2=2k^2\nu ^2$$

By analyzing (16), these solutions are suitable to all cases of the general solutions $\phi _1$–$\phi _{27}$ to ODE (15).

### *Step 4*

**Fig. 1:**
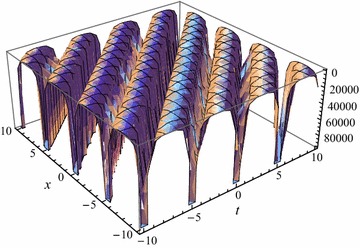
The solitary wave solutions of the exact solutions $U_1$

Acquiring the exact travelling wave solutions. By substituting (16) and the general solutions $\phi _1$–$\phi _{27}$ of ODE (15) into (14) respectively, we obtain the exact travelling wave solutions as follows:

17 $$U_j \left( \xi \right) =\frac{3c^2-k^2+k^4\delta ^2+8k^4\lambda \nu }{6k^2} +2k^2\delta \nu \phi _j+2k^2\nu ^2\phi _j^2, \quad j=1,2,\ldots ,27 $$

the solutions (17) have 27 different cases, which are expressed by the hyperbolic functions, the trigonometric functions and the rational functions, respectively. The solitary wave solutions can be obtained (see Fig. 1) when the parameters are taken as special values.

## The approximate analytic solutions of Eq. (8) based on the homotopy perturbation method

The homotopy perturbation method is proposed by He (1999), and it has successfully been applied to solve many types of linear and nonlinear functional equations. This method, which is a combination of homotopy in topology and classic perturbation techniques, provides us with a convenient way to obtain analytic or approximate solutions for a wide variety of problems arising indifferent fields. In recent years, the application of the homotopy perturbation method in nonlinear problems has been developed by scientists and engineers (He 2003, 2006; Olga 2011; Ebaid 2014; Najafi and Edalatpanah 2014).

Next, we construct the approximate analytic solutions of Eq. (8) by using the homotopy perturbation method. The $U_j(\xi )$ is a function of $\xi$ in (17), and $\xi =k\xi _1-c\xi _2$. Based on the solutions (17), we substitute $\xi =k\xi _1-c\xi _2$ into (17) and take $\xi _2=0$, then the following initial conditions of the homotopy perturbation method can be obtained.

18 $$U_j \left( \xi _1,0 \right) =\frac{3c^2-k^2+k^4\delta ^2+8k^4\lambda \nu }{6k^2} +\;2k^2\delta \nu \phi _j(\xi _1,0)+2k^2\nu ^2\phi _j^2(\xi _1,0), \quad j=1,2,\ldots ,27 $$

According to the homotopy perturbation method (He 1999), we construct the following homotopy

19 $$(1-p)(V_{\xi _2}-U_{0,\xi _2})+p(V_{11}-3V_{22}+6(V_1)^2+6VV_{11}-V_{1111})=0 $$

Equation (19) has the following form of solutions

20 $$V(\xi _1,\xi _2)=V_0(\xi _1,\xi _2)+pV_1(\xi _1,\xi _2)+p^2V_2(\xi _1,\xi _2)+\cdots ,$$

where *p* is an embedding parameter, and $V_1(\xi _1,\xi _2), V_2(\xi _1,\xi _2),\ldots$ are undetermined. In order to be convenient for computing, we choose the following initial value approximation

21 $$ U_0(\xi _1,\xi _2)=V_0(\xi _1,\xi _2)=0$$

By substituting (20) and (21) into Eq. (19) and collecting parameters $p^i(i=1,2,\ldots )$ with the aid of expansion as follows:

22 $$\begin{aligned} p^i=\left\{ \begin{array}{ll} V_{1,2}=0 &{}\quad i=1\\ -V_{1,2}+V_{2,2}+3V_{1,22}-V_{1,11}+V_{1,1111}=0 &{}\quad i=2\\ -V_{2,2}+V_{3,2}+3V_{2,22}-6(V_{1,1})^2-6V_1V_{1,11}-V_{2,11}+V_{2,1111}=0 &{}\quad i=3\\ \vdots &{}\quad \vdots \end{array} \right. \end{aligned}$$

where $V_{m,n}$ donates that $V_m(m=1,2,\ldots )$ takes derivative with respect to the $n(n=1,2)$ variant. We choose the initial conditions as follows:

23 $$ V_j(\xi _1,0)=U_j(\xi _1,0)\quad j=1,2,\ldots ,27$$

and construct the approximate analytic solutions of Eq. (8) based on the following four cases.

### *Case 1*

When $j=1$, satisfying the initial conditions as follows:

24 $$\begin{aligned} V_{i1}(\xi _1,0)=\left\{ \begin{array}{ll} \frac{3c^2-k^2-2k^4\delta ^2+8k^4\lambda \nu +3k^4\left( \delta ^2-4\lambda \nu \right) \tanh \left[ \frac{\sqrt{\delta ^2-4\lambda \nu }}{2}k\xi _1\right] ^2}{6k^2} &{}\quad i=1 \\ 0 &{}\quad i=2,3,\ldots \end{array} \right. \end{aligned}$$

the solutions can be obtained by (22) and (24) as follows:

25 $$ V_1(x,t,y)=  {} \frac{3c^2-k^2-2k^4\delta ^2+8k^4\lambda \nu +3k^4\left( \delta ^2-4\lambda \nu \right) \tanh \left[ \frac{\sqrt{\delta ^2-4\lambda \nu }}{2}k(x-t)\right] ^2}{6k^2} $$

26 $$\begin{aligned} V_2(x,t,y)&=  {} \frac{1}{16}k^4y\left( \delta ^2-4\lambda \nu \right) ^2\left( 3+33k^2\delta ^2-132k^2\lambda \nu +\left( 2-26k^2\left( \delta ^2-4\lambda \nu \right) \right) \right. \nonumber \\&\quad \times \cosh \left[ k(x-t)\sqrt{\delta ^2-4\lambda \nu }\right] +\left( -1+k^2\left( \delta ^2-4\lambda \nu \right) \right) \nonumber \\&\left. \quad \times \cosh \left[ 2k(x-t)\sqrt{\delta ^2-4\lambda \nu }\right] \right) \mathrm{sech}\left[ \frac{1}{2}k(x-t)\sqrt{\delta ^2-4\lambda \nu }\right] ^6 \end{aligned}$$

### *Remark*

Two variables $\xi _1=x-t$, $\xi _2=y$ have are substituted in (25) and (26).

Then the second-order approximate solutions of Eq. (8) can be achieved by (25) and (26)

27 $$\begin{aligned} {\mathbb {U}}_1(x,t,y)&=  {} \frac{3c^2-k^2-2k^4\delta ^2+8k^4\lambda \nu +3k^4\left( \delta ^2-4\lambda \nu \right) \tanh \left[ \frac{\sqrt{\delta ^2-4\lambda \nu }}{2}k(x-t)\right] ^2}{6k^2}\nonumber \\&\quad +\frac{1}{16}k^4y\left( \delta ^2-4\lambda \nu \right) ^2\left( 3+33k^2\delta ^2-132k^2\lambda \nu +\left( 2-26k^2\left( \delta ^2-4\lambda \nu \right) \right) \right. \nonumber \\&\quad \times \cosh \left[ k(x-t)\sqrt{\delta ^2-4\lambda \nu }\right] +\left( -1+k^2\left( \delta ^2-4\lambda \nu \right) \right) \cosh \left[ 2k(x-t)\right. \nonumber \\&\left. \left. \sqrt{\delta ^2-4\lambda \nu }\right] \right) \mathrm{sech}\left[ \frac{1}{2}k(x-t)\sqrt{\delta ^2-4\lambda \nu }\right] ^6 \end{aligned}$$

### *Case 2*

When $j=13$, satisfying the initial conditions as follows:

28 $$\begin{aligned} V_{i13}(\xi _1,0)=\left\{ \begin{array}{ll} \frac{k^2-3c^2+2k^4\delta ^2-8k^4\lambda \nu +3k^4\left( \delta ^2-4\lambda \nu \right) \tan \left[ \frac{\sqrt{4\lambda \nu -\delta ^2}}{2}k\xi _1\right] ^2}{6k^2} &{}\quad i=1\\ 0 &{}\quad i=2,3,\ldots \end{array} \right. \end{aligned}$$

the solutions can be obtained by (22) and (28) as follows:

29 $$ V_1(x,t,y)=  {} \frac{k^2-3c^2+2k^4\delta ^2-8k^4\lambda \nu +3k^4\left( \delta ^2-4\lambda \nu \right) \tan \left[ \frac{\sqrt{4\lambda \nu -\delta ^2}}{2}k(x-t)\right] ^2}{6k^2} $$

30 $$\begin{aligned} V_2(x,t,y)&=  {} \frac{1}{16}k^4y\left( \delta ^2-4\lambda \nu \right) ^2\left( 3+33k^2\delta ^2-132k^2\lambda \nu +\left( 2-26k^2\left( \delta ^2-4\lambda \nu \right) \right) \right. \nonumber \\&\quad \times \cos \left[ k(x-t)\sqrt{4\lambda \nu -\delta ^2}\right] +\left( -1+k^2\left( \delta ^2-4\lambda \nu \right) \right) \nonumber \\&\left. \quad \times \cos \left[ 2k(x-t)\sqrt{4\lambda \nu -\delta ^2}\right] \right) \sec \left[ \frac{1}{2}k(x-t)\sqrt{4\lambda \nu -\delta ^2}\right] ^6 \end{aligned}$$

Then the second-order approximate solutions of Eq. (8) can also be achieved by (29) and (30)

31 $$\begin{aligned} {\mathbb {U}}_2(x,t,y)&=  {} \frac{k^2-3c^2+2k^4\delta ^2-8k^4\lambda \nu +3k^4\left( \delta ^2-4\lambda \nu \right) \tan \left[ \frac{\sqrt{4\lambda \nu -\delta ^2}}{2}k(x-t)\right] ^2}{6k^2}\nonumber \\&\quad +\frac{1}{16}k^4y\left( \delta ^2-4\lambda \nu \right) ^2\left( 3+33k^2\delta ^2-132k^2\lambda \nu +\left( 2-26k^2\left( \delta ^2-4\lambda \nu \right) \right) \right. \nonumber \\&\quad \times \cos \left[ k(x-t)\sqrt{4\lambda \nu -\delta ^2}\right] +\left( -1+k^2\left( \delta ^2-4\lambda \nu \right) \right) \cos \left[ 2k(x-t)\right. \nonumber \\&\left. \left. \quad \times \sqrt{4\lambda \nu -\delta ^2}\right] \right) \sec \left[ \frac{1}{2}k(x-t)\sqrt{4\lambda \nu -\delta ^2}\right] ^6 \end{aligned}$$

### *Case 3*

When $j=25,$ satisfying the initial conditions as follows:

32 $$\begin{aligned} V_{i25}(\xi _1,0)=\left\{ \begin{array}{ll} \frac{8k^4\lambda \nu -k^2+3c^2+k^4\delta ^2}{6k^2}+\frac{2k^2\delta ^2\omega (-\cosh [\delta k\xi _1]+\sinh [\delta k\xi _1])}{\left( \omega +\cosh [\delta k\xi _1]-\sinh [\delta k\xi _1]\right) ^2} &{}\quad i=1\\ 0 &{}\quad i=2,3,\ldots \end{array} \right. \end{aligned}$$

the solutions can be obtained by (22) and (32) as follows:

33 $$ V_1(x,t,y)=  {} \frac{8k^4\lambda \nu -k^2+3c^2+k^4\delta ^2}{6k^2} +\frac{2k^2\delta ^2\omega \left( \sinh [\delta k(x-t)]-\cosh [\delta k(x-t)]\right) }{1\left( \omega +\cosh [\delta k(x-t)]-\sinh [\delta k(x-t)]\right) ^2}$$

34 $$\begin{aligned} V_2(x,t,y)&=  {} \frac{2k^4\delta ^4\omega y}{\left( \omega +\cosh [k(x-t)\delta ]-\sinh [k(x-t)\delta ]\right) ^6}\left( 6\omega ^2+66k^2\delta ^2\omega ^2\right. \nonumber \\&\quad -2\left( -1+13k^2\delta ^2\right) \omega \left( 1+\omega ^2\right) \cosh [k(x-t)\delta ]+\left( -1+k^2\delta ^2\right) \left( 1+\omega ^4\right) \nonumber \\&\quad \times \cosh [2k(x-t)\delta ]-2\omega \sinh [k(x-t)\delta ]+26k^2\delta ^2\omega \sinh [k(x-t)\delta ]\nonumber \\&\quad +2\omega ^3\sinh [k(x-t)\delta ]-26k^2\delta ^2\omega ^3\sinh [k(x-t)\delta ]+\sinh [2k(x-t)\delta ]\nonumber \\&\quad -k^2\delta ^2\sinh [2k(x-t)\delta ]-\omega ^4\sinh [2k(x-t)\delta ]\nonumber \\&\left. \quad +k^2\delta ^2\omega ^4\sinh [2k(x-t)\delta ]\right) (\cosh [3k(x-t)\delta ]-\sinh [3k(x-t)\delta ]) \end{aligned}$$

Then the second-order approximate solutions of Eq. (8) can also be achieved by (33) and (34)

35 $$\begin{aligned} {\mathbb {U}}_3(x,t,y)&=  {} \frac{8k^4\lambda \nu -k^2+3c^2+k^4\delta ^2}{6k^2}+\frac{2k^2\delta ^2\omega (\sinh [\delta k(x-t)]-\cosh [\delta k(x-t)])}{\left( \omega +\cosh [\delta k(x-t)]-\sinh [\delta k(x-t)]\right) ^2}\nonumber \\&\quad +\frac{2k^4\delta ^4\omega y}{\left( \omega +\cosh [k(x-t)\delta ]-\sinh [k(x-t)\delta ]\right) ^6}\left( 6\omega ^2+66k^2\delta ^2\omega ^2\right. \nonumber \\&\quad -2\left( -1+13k^2\delta ^2\right) \omega \left( 1+\omega ^2\right) \cosh [k(x-t)\delta ]+\left( -1+k^2\delta ^2\right) \left( 1+\omega ^4\right) \nonumber \\&\quad \times \cosh [2k(x-t)\delta ]-2\omega \sinh [k(x-t)\delta ]+26k^2\delta ^2\omega \sinh [k(x-t)\delta ]\nonumber \\&\quad +2\omega ^3\sinh [k(x-t)\delta ]-26k^2\delta ^2\omega ^3\sinh [k(x-t)\delta ]+\sinh [2k(x-t)\delta ]\nonumber \\&\quad -k^2\delta ^2\sinh [2k(x-t)\delta ]-\omega ^4\sinh [2k(x-t)\delta ]\nonumber \\&\left. \quad +k^2\delta ^2\omega ^4\sinh [2k(x-t)\delta ]\right) (\cosh [3k(x-t)\delta ]-\sinh [3k(x-t)\delta ]) \end{aligned}$$

### *Case 4*

When $j=27$, satisfying the initial conditions as follows:

36 $$\begin{aligned} V_{i27}(\xi _1,0)=\left\{ \begin{array}{ll} \frac{-1}{6}+\frac{c^2}{2k^2}+\frac{k^2\delta ^2}{6}+\frac{4}{3}k^2\lambda \nu +\frac{2k^2\nu ^2}{(c+\nu k\xi _1)^2}-\frac{2k^2\delta \nu }{c+\nu k\xi _1} &{}\quad i=1\\ 0 &{}\quad i=2,3,\ldots \end{array} \right. \end{aligned}$$

the solutions can be obtained by (22) and (36) as follows:

37 $$\begin{aligned} V_1(x,t,y)&=  {} \frac{-1}{6}+\frac{c^2}{2k^2}+\frac{k^2\delta ^2}{6}+\frac{4}{3}k^2\lambda \nu +\frac{2k^2\nu ^2}{[c+\nu k(x-t)]^2}-\frac{2k^2\delta \nu }{c+\nu k(x-t)} \end{aligned}$$

38 $$\begin{aligned} V_2(x,t,y)&=  {} -\frac{4k^4y\nu ^3\left[ c^3\delta +3c^2(\delta k(x-t)-1)\nu \right] }{[c+\nu k(x-t)]^6}-\frac{3ck\left[ -2(x-t)-4k\delta +k\delta (x-t)^2\right] \nu ^2}{[c+\nu k(x-t)]^6}\nonumber \\&\quad -\frac{k^2y\left[ k\delta (x-t)^3-12k\delta (x-t)-3(x-t)^2+60\right] \nu ^3}{[c+\nu k(x-t)]^6} \end{aligned}$$

Then the second-order approximate solutions of Eq. (8) can also be achieved by (37) and (38)

39 $$\begin{aligned} {\mathbb {U}}_4(x,t,y)&=  {} -\frac{1}{6}+\frac{c^2}{2k^2}+\frac{k^2\delta ^2}{6}+\frac{4}{3}k^2\lambda \nu +\frac{2k^2\nu ^2}{[c+\nu k(x-t)]^2}-\frac{2k^2\delta \nu }{c+\nu k(x-t)}\nonumber \\&\quad -\frac{4k^4y\nu ^3\left[ c^3\delta +3c^2(\delta k(x-t)-1)\nu \right] }{[c+\nu k(x-t)]^6}-\frac{3ck\left[ -2(x-t)-4k\delta +k\delta (x-t)^2\right] \nu ^2}{[c+\nu k(x-t)]^6}\nonumber \\&\quad -\frac{k^2y\left[ k\delta (x-t)^3-12k\delta (x-t)-3(x-t)^2+60\right] \nu ^3}{[c+\nu k(x-t)]^6} \end{aligned}$$

**Fig. 2:**
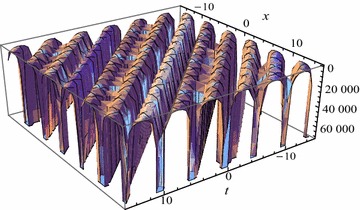
The exact solutions $U_{13}$ for $k=9$, $c=25$, $\alpha =4.25$, $\delta =3$, $\lambda =1$, $\nu =2$

**Fig. 3:**
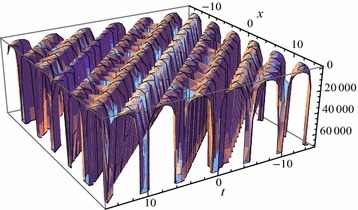
The approximate analytic solutions ${\mathbb {U}}_2$ for $k=9$, $c=25$, $\alpha =4.25$, $\delta =3$, $\lambda =1$, $\nu =2$

Figures 2 and 3 show the exact solutions (17) ($j=13$) and the second-order approximate solutions (31) based on the homotopy perturbation method of Eq. (8) respectively when the parameters are regarded as proper values. Table 1 shows the error comparison between the solutions (17) ($j=1$) and (27) when $k=0.1$, $c=0.2$, $\delta =3$, $\lambda =1$, $\nu =2$. According to the figure and table, the exact property of the homotopy perturbation method has been showed successfully.

**Table 1:** The error comparison between $U_{1}$ and ${\mathbb{U}}_1$ at y=0.2

t	Error				
	x				
	0.01	0.02	0.03	0.04	0.05
0.05	2.70070 × 10^−6^	2.80067 × 10^−6^	2.90063 × 10^−6^	3.00058 × 10^−6^	3.10053 × 10^−6^
0.10	2.20078 × 10^−6^	2.30077 × 10^−6^	2.40076 × 10^−6^	2.50074 × 10^−6^	2.60072 × 10^−6^
0.15	1.70074 × 10^−6^	1.80076 × 10^−6^	1.90077 × 10^−6^	2.00078 × 10^−6^	2.10078 × 10^−6^
0.25	2.00189 × 10^−7^	3.00238 × 10^−7^	4.00286 × 10^−7^	5.00333 × 10^−7^	6.00378 × 10^−7^
0.30	1.60072 × 10^−6^	8.00465 × 10^−7^	9.00506 × 10^−7^	1.00054 × 10^−6^	1.10058 × 10^−6^

## Conclusion

In this paper, we studied that construct the exact solutions and the approximate analytic solutions of NLPDE by using the *Lie* symmetry, the extended tanh method and the homotopy perturbation method. Specifically, we have constructed the abundant exact travelling wave solutions and approximate analytic solutions of the (2 + 1)-dimensional KP equation by using the above-mentioned three methods and obtained the high-precision approximate solutions by error analysis.

*Lie* symmetry, the extended tanh method and the homotopy perturbation method are effective methods which applied to solve PDEs. Hence, comprehensive use of them will advance their availability. The Wu-differential characteristic set algorithm is a key factor which influence the calculating the symmetry of PDEs. At present, combining the Wu-differential characteristic set algorithm, symmetry method and others to solve NLPDE has been regarded as a hot research topic and widened the application of symmetry and the Wu-differential characteristic set algorithm. This investigation is valuable in advanced research and development.

## Abbreviations

NLPDE: nonlinear partial differential equations
PDEs: the partial differential equations
ODE: ordinary differential equation
